# P34 The value of combined ophthalmology and rheumatology review- an unmet need

**DOI:** 10.1093/rap/rkab068.033

**Published:** 2021-10-19

**Authors:** Arumugam Moorthy, Priti Kulkarni, V Patel, Periyasamy Kumar

**Affiliations:** 1 Rheumatology Department, Leicester Royal Infirmary, Leicester, United Kingdom; 2 Ophthalmology Department, Leicester Royal Infirmary, Leicester, United Kingdom

## Abstract

**Case report - Introduction:**

Rheumatic diseases manifest in different specialties including ophthalmology. Presentation with eye symptoms may be a sight-threatening emergency such as GCA. Other inflammatory symptoms such as uveitis present to the Eye Casualty frequently, which need prompt rheumatologist input for a holistic management.

**Case report - Case description:**

We present an interesting case with multiple learning points for both rheumatologists as well as ophthalmologists.

We present a case of a 73-year-old Caucasian lady who initially presented to the Eye Department. She was diagnosed as having bilateral uveitis by the ophthalmologist with an interest in uveitis. She had various investigations which showed positive HLA-B27 status, ENA, ANA, serum ACE level were normal; however, she was found to have raised immunoglobulins and plasma viscosity. She was treated with steroid eye drops and intravitreal dexamethasone implant in both eyes. She is a steroid responder and unfortunately developed glaucoma which needed two surgical procedures. She developed chronic cystoid macular oedema in Left eye.

She did not have any symptoms of psoriasis or gut symptoms suggestive of inflammatory bowel disease. She did report symptoms of inflammatory bowel disease. She had an MRI of axial spine as per AS protocol, which confirmed inflammatory spondyloarthropathy. She was diagnosed to have non-radiographic spondyloarthropathy which is managed by simple anti-inflammatories. Her BASDAI is less than 3 and does not qualify for biologic treatment for her axial spondyloarthropathy. Her main symptoms were ocular, which is very active and she is not able to escalate to biologic therapy.

**Case report - Discussion:**

This is a patient presenting with late-onset inflammatory back pain without any articular or extra-articular activity. Her main manifestation is spectrum of spondyloarthropathy with uveitis; however, we could not escalate her to biologics. She has been on topical, intraocular and oral steroids which has led on to complications. The combined clinic between rheumatologists and ophthalmologists is key in managing such patients.

**Case report - Key learning points:**

1. Recurrent uveitis in HLA-B27 patients needs to be reviewed in ophthalmology combined services with rheumatologists to establish the diagnosis of SpA
2. Delay of diagnosis of SpA can be reduced by establishing combined uveitis/SpA services within the NHS widely

